# Supplementary material for: Using targeted sequencing and TaqMan approaches to detect acaricide (bifenthrin, bifenazate, and etoxazole) resistance associated SNPs in Tetranychus urticae collected from peppermint fields and hop yards
Source: PLoS One. 2023 Mar 23;18(3):e0283211. doi: 10.1371/journal.pone.0283211 (PMC10035822; doi:10.1371/journal.pone.0283211)
Supplement: S1 Table — (DOCX) [file pone.0283211.s001.docx]

Supplemental Table 1: Primers and probes for the TaqMan genotyping assay and primers for the multiplex PCR assay

| Assay | Mutation | Forward Primer Seq. | Reverse Primer Seq. | Reporter 1 Sequence | Reporter 2 Sequence | Accession # |
| --- | --- | --- | --- | --- | --- | --- |
| TaqMan | F1538I | ACAACCAGTTTATGAAAATAGTATTCTGATGTACTT | CACCTCCTTTCTTTTTTTGTTCATTAAAATTATCAATAATG | ATTTTTGGCTCTTTTTTCACAC | TTTTGGCTCTTTTATCACAC | JN881331.1 |
| TaqMan | G126S | AGGATCCGCTTTTATTGGGT | AGTAATAACTGTTGCTCCCCAAAAAGAT | ATGTTTTACCTTGAGGACAAA | TGTTTTACCTTGAAGACAAA | EU556749.1 |
| TaqMan | I1017F | GCTTCATCCACAAGAGTTTCACTGT | ACGTTCAAGTTGACCAGAGAATAGAT | ATTTCCTTTCGATTCCATG | ATTTCCTTTCGTTTCCATG | tetur03g08510 |
| Multiplex | F1538I | AACAACCAGTTTATGAAAATAGTATTCTGATGTACTTA | CACCTCCTTTCTTTTTTTGTTCATTAAAATTATCAATAATG | NA | NA | JN881331.1 |
| Multiplex | G126S | AGGATCCGCTTTTATTGGGTATG | TCAACGGAAAATCTTCCCCAAAC | NA | NA | EU556749.1 |
| Multiplex | I1017F | TGCTTCATCCACAAGAGTTTCA | ACGTTCAAGTTGACCAGAGAATAG | NA | NA | tetur03g08510 |
