# Supplementary material for: Using targeted sequencing and TaqMan approaches to detect acaricide (bifenthrin, bifenazate, and etoxazole) resistance associated SNPs in Tetranychus urticae collected from peppermint fields and hop yards
Source: PLoS One. 2023 Mar 23;18(3):e0283211. doi: 10.1371/journal.pone.0283211 (PMC10035822; doi:10.1371/journal.pone.0283211)
Supplement: Attachment — Submitted filename: Review report PONE-D-22-09155.docx [file pone.0283211.s002.docx]

Review report PONE-D-22-09155: Detection of bifenthrin, bifenazate, etoxazole resistance in *Tetranychus urticae* collected from peppermint fields and hop yards using targeted sequencing and TaqMan approaches in pooled DNA samples

*Tetranychus urticae* is an important pest and recorded on more than 1,000 different plant species infested worldwide. Although several methods have been used frequently, farmers mostly rely on chemical pesticides against *T. urticae*. But the acaricide resistance can develop through multiple mechanisms, including enhanced metabolic breakdown of acaricides, target site insensitivity, and behavioral resistance. The authors examined the use of a TaqMan qPCR-based approach to determine acaricide resistance genotypes in field-collected populations of *T. urticae* from peppermint fields and hop yards in the Pacific Northwest of the United States and confirmed the results with a multiplex targeted sequencing. Although the MS is well written, the following points need to be addressed.

L 26-27: Multiple acaricides are currently registered for control including bifenazate, bifenthrin, and extoxazole > Multiple acaricides including bifenazate, bifenthrin, and extoxazole are currently registered for control *T. urticae*.

L 29-30: Not clear. What is integrated pest management tools?

L 31-34: The authors mentioned “The detection of these mutations through TaqMan qPCR has been suggested as a practical, quick, and reliable tool to inform agricultural producers of acaricide resistance phenotypes present within their fields and have potential utility for making appropriate acaricide application and integrated pest management decisions”—Here is the major concern that the authors emphasis the TaqMan qPCR throughout the MS to determine acaricide resistance genotypes. This is not a new method to determine to detect different chemical resistance status of spider mites. It would be better to compare with other method and confirm the accuracy to determine resistance genotypes against specific chemicals.

L 71-72: Any reference(s).

L 77-79: Pls rephrase

L 197: Why the author performs the median lethal dose assay only for bifenazate?

L 277-280: Add reference(s)

L 281-284: If the method is already well-known molecular tool for growers to detect different chemical resistance status of *T. urticae*, what is the novelty of present research. (Pls check reference 19, 20)

None of the scientific name is Italic in reference section.
