# Supplementary material for: Using targeted sequencing and TaqMan approaches to detect acaricide (bifenthrin, bifenazate, and etoxazole) resistance associated SNPs in Tetranychus urticae collected from peppermint fields and hop yards
Source: PLoS One. 2023 Mar 23;18(3):e0283211. doi: 10.1371/journal.pone.0283211 (PMC10035822; doi:10.1371/journal.pone.0283211)
Supplement: Attachment — Submitted filename: Response to Reviewers 9_15_22.docx [file pone.0283211.s003.docx]

**Cover Letter**

Dear Dr. Hamim and Reviewers

Thank you very much for your review and consideration of our manuscript “PONE-D-22-09155, Title: Detection of bifenthrin, bifenazate, etoxazole resistance in Tetranychus urticae collected from peppermint fields and hop yards using targeted sequencing and TaqMan approaches in pooled DNA samples”, submitted for review in PLOS ONE. We understand the amount of work and effort that the editor and reviewers have taken to improve this manuscript and appreciate your feedback and comments.

We took the comments from the reviewers very seriously. The comments from the reviewers and the editor were helpful in improving our manuscript. Please find enclosed our corrections and comments to the reviewers, on a point-by-point basis. Reviewer and editor comments are in font color black and response to reviewers’ comments are in blue. We feel this manuscript has benefited from the peer-review process and look forward to any further suggestions for improvement if they arise. The corresponding author has left the institution in which the research was conducted and, as such, no follow up research or laboratory studies can be conduct. We believe that this does not impact the findings of the investigation and its utility to the agricultural community.

Sincerely,

The Authors

Justin Clements (corresponding author), Ph.D.

[Jclements3323@gmail.com](mailto:Jclements3323@gmail.com)

Funding section as requested by the journal is listed in the cover letter below.

This research was supported by funding from United States Department of Agriculture Specialty Crop Initiative 2021-51181-35901, awarded to DW and JC, a Mint Industry Research Council grant awarded to DW and JC, a Washington Mint Commission grant awarded to DW, and startup funds awards from the University of Idaho to JC. There was no additional external funding received for this study.

**Journal Requirements:**

1. Please ensure that your manuscript meets PLOS ONE's style requirements, including those for file naming.

We have reviewed the naming guidelines for files. We have changed Figure 1 to read Fig 1 in both the manuscript and the file name. We have changed Supplemental File 1 to read S1 file in both the manuscript and the file name.

2. Thank you for stating in your Funding Statement:

This research was supported in part by funding from United States Department of Agriculture Specialty Crop Initiative 2021-51181-35901, awarded to DW and JC, a Mint Industry Research Council grant awarded to DW and JC, and a Washington Mint Commission grant awarded to DW and JC.

Please provide an amended statement that declares *all* the funding or sources of support (whether external or internal to your organization) received during this study, as detailed online in our guide for authors at http://journals.plos.org/plosone/s/submit-now. Please also include the statement “There was no additional external funding received for this study.” in your updated Funding Statement.

Please include your amended Funding Statement within your cover letter. We will change the online submission form on your behalf.

We have removed the funding section from the manuscript and have included it in our cover letter. The funding section now reads “This research was supported by funding from United States Department of Agriculture Specialty Crop Initiative 2021-51181-35901, awarded to DW and JC, a Mint Industry Research Council grant awarded to DW and JC, a Washington Mint Commission grant awarded to DW, and startup funds awards from the University of Idaho to JC. There was no additional external funding received for this study.”

3. Thank you for stating the following in the Acknowledgments Section of your manuscript:

This research was supported in part by funding from United States Department of Agriculture Specialty Crop Initiative 2021-51181-35901, awarded to DW and JC, a Mint Industry Research Council grant awarded to DW and JC, and a Washington Mint Commission grant awarded to DW.

However, funding information should not appear in the Acknowledgments section or other areas of your manuscript. We will only publish funding information present in the Funding Statement section of the online submission form.

Please remove any funding-related text from the manuscript and let us know how you would like to update your Funding Statement. Currently, your Funding Statement reads as follows:

This research was supported in part by funding from United States Department of Agriculture Specialty Crop Initiative 2021-51181-35901, awarded to DW and JC, a Mint Industry Research Council grant awarded to DW and JC, and a Washington Mint Commission grant awarded to DW and JC.

Please include your amended statements within your cover letter; we will change the online submission form on your behalf.

We have removed the funding section from the manuscript and have included it in our cover letter. The funding section now reads “This research was supported by funding from United States Department of Agriculture Specialty Crop Initiative 2021-51181-35901, awarded to DW and JC, a Mint Industry Research Council grant awarded to DW and JC, a Washington Mint Commission grant awarded to DW, and startup funds awards from the University of Idaho to JC. There was no additional external funding received for this study.”

4. Your ethics statement should only appear in the Methods section of your manuscript. If your ethics statement is written in any section besides the Methods, please move it to the Methods section and delete it from any other section. Please ensure that your ethics statement is included in your manuscript, as the ethics statement entered into the online submission form will not be published alongside your manuscript.

We have moved the ethics section to the Methods.

5. We note that Figure 1 in your submission contain [map/satellite] images which may be copyrighted. All PLOS content is published under the Creative Commons Attribution License (CC BY 4.0), which means that the manuscript, images, and Supporting Information files will be freely available online, and any third party is permitted to access, download, copy, distribute, and use these materials in any way, even commercially, with proper attribution. For these reasons, we cannot publish previously copyrighted maps or satellite images created using proprietary data, such as Google software (Google Maps, Street View, and Earth). For more information, see our copyright guidelines: http://journals.plos.org/plosone/s/licenses-and-copyright.

We require you to either (1) present written permission from the copyright holder to publish these figures specifically under the CC BY 4.0 license, or (2) remove the figures from your submission:

a. You may seek permission from the original copyright holder of Figure 1 to publish the content specifically under the CC BY 4.0 license.

We recommend that you contact the original copyright holder with the Content Permission Form (http://journals.plos.org/plosone/s/file?id=7c09/content-permission-form.pdf) and the following text:

“I request permission for the open-access journal PLOS ONE to publish XXX under the Creative Commons Attribution License (CCAL) CC BY 4.0 (http://creativecommons.org/licenses/by/4.0/). Please be aware that this license allows unrestricted use and distribution, even commercially, by third parties. Please reply and provide explicit written permission to publish XXX under a CC BY license and complete the attached form.”

Please upload the completed Content Permission Form or other proof of granted permissions as an "Other" file with your submission.

In the figure caption of the copyrighted figure, please include the following text: “Reprinted from [ref] under a CC BY license, with permission from [name of publisher], original copyright [original copyright year].”

b. If you are unable to obtain permission from the original copyright holder to publish these figures under the CC BY 4.0 license or if the copyright holder’s requirements are incompatible with the CC BY 4.0 license, please either i) remove the figure or ii) supply a replacement figure that complies with the CC BY 4.0 license. Please check copyright information on all replacement figures and update the figure caption with source information. If applicable, please specify in the figure caption text when a figure is similar but not identical to the original image and is therefore for illustrative purposes only.

The following resources for replacing copyrighted map figures may be helpful:

Fig 1 is the author’s original work and contains no copyrighted material. The map was constructed with QGIS, a free and open-source GIS software, using state boundary shapefiles sourced from the US Census Bureau, which are freely provided to the public online and are not copyrighted.

**Reviewer #1**

Abstract

The abstract need to rewritten with key information.

Rewrite the statement “The results suggest the TaqMan approach accurately genotypes T. urticae populations collected from agricultural fields” for better understanding.

We have added additional information into the abstract regarding key findings and have reworded the above sentence.

Introduction

The molecular mechanism for acaricide resistance may be improved from following latest references

1. https://doi.org/10.1016/j.pestbp.2021.104985

2. https://doi.org/10.11158/saa.26.8.10

3. https://doi.org/10.1186/s13071-020-04227-7

We have added two of the above citations to improve the manuscript. We refrained from using the citation from Cossío-Bayúgar, as it was focused on *Rhipicephalus microplus* (tick parasite).

Materials & Method

1. Provide the reference for CethylTrimethyl Ammonium Bromide (CTAB) method and company details for Zymo Research Quick-DNA Tissue/132 Insect Microperp kit.

We have added the company name for both the CTAB reagent and Zymo Kit.

2. Line no 135, specify type and wavelength of UV light

We have added the wavelength, which was 254 nm

3. DNA extraction procedure may be shortened by providing suitable reference and the modification made in protocol. Similarly, the kit method shortened by citing manufacturers’ instruction.

In the methods we have added the company name and that the CTAB reaction was a modified reaction (which is the reason why we have included a detailed procedure).

4. Line no 198 replicate or replication

Replicate is the correct term

Result & Discussion

1. Authors should explain the reasons for the samples did not amplify in Table 3 in results.

Sample 12 did not amplify in the Illumina reaction. We are unaware of why this sample was not able to be amplified, as the reaction was sent to a third party for amplification. Multiple reasons could be behind the non-amplification on the Illumina run. Because this amplification was conducted by a third party, we do not want to speculate on the reasoning behind why sample 12 did not amplify.

**Reviewer #2**

Dear Dr. Islam Hamim

Manuscript #: PONE-D-22-09155

Title: Detection of bifenthrin, bifenazate, etoxazole resistance in Tetranychus urticae collected from peppermint fields and hop yards using targeted sequencing and TaqMan approaches in pooled DNA samples

Whether the use of using two different molecular approaches to determine the resistance genotypes present in T. urticae from collected field are the main question in this study. For this aim, Authors examined the use of a TaqMan qPCR-based approach to determine acaricide resistance genotypes in field-collected populations of T. urticae from peppermint fields and hop yards in the Pacific Northwest of the United States and confirmed the results with a multiplex targeted sequencing. The results shown the TaqMan approach accurately genotypes T. urticae populations collected from agricultural fields. However, as the researchers of this method stated, it needs to be further developed for the correct determination of resistance in T. urticae. Therefore, this manuscript contains content of applicable value. This manuscript is appropriate to be published in the PLOS ONE after some corrections that I have mentioned below:

1. In "Running Title", acaricide should be used instead of miticide.

Changed as suggested

2. The abstract should be reviewed and some conclusions should be given.

We have added conclusion to the abstract as suggested

3. Line 122 “dry” should be removed.

Removed as suggested

4. Information about the laboratory population of T. urticae should be given in the material and method.

We have added, “A laboratory population of *T. urticae* was used as a baseline reference for the LD50 assays. This population represents a historically susceptible population that has been housed at the IAREC center in Processer Washington for multiple generations, has not been subjected to any chemical exposure over those generations, and has been raised on beans.” To describe the susceptible laboratory population

5. In DNA isolation from Tetranychus urticae section, the mite stage used in DNA isolation should be specified.

We have added, “DNA extractions were conducted on pooled motile mites encompassing multiple life stages.”

6. In the determination of the bifenazate L50 value, 4 doses were used, except for the control. At least 5 doses were required to determine reliable LC50 (Yu,2015, Simon, J. Y. (2015). The toxicology and biochemistry of insecticides. CRC press.).

Yu (2015) suggests, “The animals available for testing are divided at random into several groups, usually six. One of these is used as the control group…” We agree that more doses would refine the estimate but there is no defined number of doses required for LD50 assessment. Many studies have utilized less than 6 doses for LD50 studies and the tested rates were the available rate at the time of the assay.

7. In the medium lethal dose assay section, how bifenazate was applied to mites should be written clearly.

We have added that treatments were sprayed onto the leaf discs using a potter spray tower in the methods section

8. Line 351, mint should be written instead of mites.

We have changed mites to mint as suggested. Thank you for the correction.

**Reviewer #3:**

Detection of bifenthrin, bifenazate, etoxazole resistance in Tetranychus urticae collected from peppermint fields and hop yards using targeted sequencing and TaqMan approaches in pooled DNA samples

Authors Shumate et al. presented a study to detect SNPs that are associated with acaracide resistance in Tetranychus urticae by using targeted sequencing and TaqMan approaches. Their results showed that both diagnostic methods exhibited consistent results, but TaqMan approach may have potential to be used in the field since this method is relatively easier and taking less time compared to targeted sequencing. However, when authors interpreted the results of phenotypes and genotypes, more discussion is needed. G126S is a mutation associated with low level of bifenezate resistance (several citations needed here) but recent study (reference 24) suggested that G126S may not link to bifenezate resistance. Besides target site insensitivity (mutations on the target proteins that have been functional studied), other mechanisms such as enhanced metabolic detoxification (P450s, esterases, GSTs) or sequestrations (ABC transporters) may also play roles in acaricide resistance. Authors only cited 24 papers in their manuscript. More previous studies associated with this topic should be discussed when authors interpreted their results and pointed out future directions.

We have added more citation as requested by reviewers throughout the manuscript and further provide more interpretation of the results.

1. Change to a more specific title: Using targeted sequencing and TaqMan approaches to detect acaricide resistance associated SNPs in Tetranychus urticae collected from peppermint fields and hop yards

Changed as suggested

2. Material and Method

Page 11, line 115: detailed information of these 13 field-collected populations (location-latitude and longitude, collection time) and the lab susceptible population should be listed in the main text or in a supplementary table.

We have provided more information about the lab susceptible population within the methods. We are unable to provide the exact field location due to confidentiality agreements with the growers that we work with.

3. Material and Method

Medium lethal dose assay: not very clear. Some details missed, such as which method was used for pesticide treatment? Any machine used for bioassay? Please cite some previous studies/publications or provide more details here.

We have added more information about the method of treating the leaf disks. “Treatments were sprayed onto the leaf surface with a potter spray tower. Ten gravid adult female *T. urticae* were placed on each leaf disk. Each bioassay arena for each of the tested rates was treated with 2 ml of the dilute solution. After 24 hours mites, were evaluated for mortality.”

4. Table 1 and Table 2 are same as the Table 3. Suggested to delete Table 1&2 and only leave the Table 3

We have removed table 2, as it has the same data incorporated into table 3. However, Table 1 has unique and important information.

5. Table 1 and Table 3: Population 1&2 in I1017F (Tagman) shows “Resistance”, however, other populations showed “wildtype” or “heterozygous”. Suggest changing “Resistance” to “heterozygous” or “homozygous”

We agree that this is confusing and have added a foot note to both tables. The reason why we do not list the resistance population as homozygous is that homozygous could refer to either wildtype or the resistant population, depending on the genotype.

6. Table 4. The bioassay data only exhibited resistance to bifenezate but did not include other two acaricides. Since the purpose of this experiment is to show if phenotype and genotype matches. G126S is not a good predictor for bifenezate resistance, then other two bioassay data should be more important. Authors should add data or at least discuss other possible reasons (previous bioassay data, other resistance mechanisms besides target site insensitivity, etc).

We agree that the additional bioassays would have been beneficial to this manuscript. Unfortunately, the data was not generated for the other two acaricide and cannot be reported on. We have added a section into the discussion regarding the other modes of detoxification could be responsible for miticide resistance as suggested by the reviewer.

**Reviewer #4:**

Review report PONE-D-22-09155: Detection of bifenthrin, bifenazate, etoxazole resistance in Tetranychus urticae collected from peppermint fields and hop yards using targeted sequencing and TaqMan approaches in pooled DNA samples

Tetranychus urticae is an important pest and recorded on more than 1,000 different plant species infested worldwide. Although several methods have been used frequently, farmers mostly rely on chemical pesticides against T. urticae. But the acaricide resistance can develop through multiple mechanisms, including enhanced metabolic breakdown of acaricides, target site insensitivity, and behavioral resistance. The authors examined the use of a TaqMan qPCR-based approach to determine acaricide resistance genotypes in field-collected populations of T. urticae from peppermint fields and hop yards in the Pacific Northwest of the United States and confirmed the results with a multiplex targeted sequencing. Although the MS is well written, the following points need to be addressed.

L 26-27: Multiple acaricides are currently registered for control including bifenazate, bifenthrin, and extoxazole > Multiple acaricides including bifenazate, bifenthrin, and extoxazole are currently registered for control T. urticae.

Changed.

L 29-30: Not clear. What is integrated pest management tools?

Removed sentence to avoid any confusion

L 31-34: The authors mentioned “The detection of these mutations through TaqMan qPCR has been suggested as a practical, quick, and reliable tool to inform agricultural producers of acaricide resistance phenotypes present within their fields and have potential utility for making appropriate acaricide application and integrated pest management decisions”—Here is the major concern that the authors emphasis the TaqMan qPCR throughout the MS to determine acaricide resistance genotypes. This is not a new method to determine to detect different chemical resistance status of spider mites. It would be better to compare with other method and confirm the accuracy to determine resistance genotypes against specific chemicals.

We agree that this method has been suggested as a tool previously. The authors wanted to make sure that this was clear. However, it has not been vetted in agriculturally relevant fields in a manner that would be practical to hops and mint growers. In this investigation, we worked with growers to sample fields for mites and processed the mites in a similar method (pooled samples) that a grower would encounter. This investigation was meant as a first step to further investigate TaqMan approaches to genotyping field populations of mites and additional studies should conducted to compare classical methods of resistance detection to TaqMan qPCR.

L 71-72: Any reference(s).

We have added a reference to support this section.

L 77-79: Pls rephrase

We have rephrased, “These polymorphisms are non-synonymous and may be the result of natural selection when they result in beneficial traits (16).”

L 197: Why the author performs the median lethal dose assay only for bifenazate?

We agree that the additional bioassays would have been beneficial to this manuscript. Unfortunately, the data was not generated for the other two acaricide and cannot be reported on. Bifenazate at the time was the only acaricide for which we examined LD_50_ and we are no longer able to perform these studies.

L 277-280: Add reference(s)

We have added references for this section

L 281-284: If the method is already well-known molecular tool for growers to detect different chemical resistance status of T. urticae, what is the novelty of present research. (Pls check reference 19, 20)

In line 281 we suggested, “The TaqMan qPCR has been suggested as a molecular tool for growers to monitor resistance status of T. urticae (21,22) and we set out to determine if a TaqMan-based approach would be a quick and reliable tool for growers to provide information regarding resistant phenotypes of local populations.” While it has been suggested for this purpose, it has not been field tested or examined thoroughly.

None of the scientific name is Italic in reference section.

Changed as suggested

**Reviewer #5:**

Clements et al. used TaqMan qPCR/Multiplex targeted sequencing to determine acaricide resistance genotypes in field populations of Tetranychus urticae in the Northwest of the USA. Their results indicate that TaqMan approach accurately genotypes T. urticae populations but according to the authors interpretation of results is challenging.

The difficult interpretation of the results might partly be caused by the fact that G126S in cytb (used by the the authors for bifenazate resistance screening) is no longer considered as a bifenazate resistance mutation (Xue et al. 2021, PMS). The authors do acknowledge Xue et al. 2021 but only in the penultimate paragraph of their manuscript. I think the authors should be more upfront about this, and change abstract/introduction accordingly (i.e. that their results confirm findings of Xue et al. 2021). They could change title to "Detection of acaricide resistance in Tetranychus urticae collected from ....", mention in the abstract that "G126S is not a good bifenzate resistance indicator" ; also rephrase line 88: "This mutation has been shown to confer resistance" and line 107: "G126S is a well-defined marker of resistance", and change line 211 to "TaqMan quantitive PCR detection of target-site mutations". Wihthout these changes, I believe the manuscript will contribute to the misinterpretation of the role of the G126S in bifenazate resistance and cannot be accepted for publication.

We agree with the reviewer and have changed the title of the manuscript. We mention that G126S is not a good indicator of resistance in the introduction and abstract. We feel that findings of this manuscript support the work that was done by Xue et al. 2021. Much of our work was conducted significantly before Xue et al. manuscript was published and feel how it is written is appropriate manner and supports their findings.

Other comments:

line 90: I1017F in Chs1 was also shown to confer resistance against hexythiazox and clofentezine (10.1016/j.ibmb.2014.05.004). Authors should include this reference in the introduction

Added as suggested

DNA extraction methods: The authors do mention two extraction methods (Zymo kit vs CTAB) but do not compare results between the two methods (e.g. 260/280 values or yield?). Author should include this comparison in the main text of the manuscript.

We have added additional information comparing the two extraction methods in the results as suggested by the reviewer.

Table 3 shows significant overlap with Table 1/2. Authors should combine these three tables into one table.

We have removed table 2, as it has much of the same information as table 3. We have left table 1, as it has unique and necessary information.

line 279: I1017F mutation has also been screened in T. urticae populations using resitriction digest/CT method (j.pestbp.2017.04.003); authors should integrate this reference in the Discussion or Introduction

Added as suggested

line 321: other VGSC mutations have also been implicated in bifenthrin resistance (L1024V) and non target site

mediated resistance mechanisms are also involved in bifenthrin resistance (see Riga et al. 2014, 10.1038/s41598-017-09054-y), which cannot be detected by the Taqman assay developed in this study. Author should integrate this reference in the Discussion section.

Added as suggested in the discussion.

**Reviewer #6:**

The manuscript “Detection of bifenthrin, bifenazate, etoxazole resistance in Tetranychus urticae collected from peppermint fields and hop yards using targeted sequencing and

TaqMan approaches in pooled DNA samples” by Shumate et al., describes the analysis of 13 T. urticae populations for the bifenthrin, bifenazate, etoxazole resistance. They used two methods to assess target site mutations that were reported to be associated with resistance to these pesticides, TaqMan and Multiplex targeted-sequencing (not for bifenazate), and have determined the actual resistance status of collected field mite populations. They checked the correlation/predictability between these parameters and found that target SNPs were congruently detected with either of the sequencing methods, but that genotyping information did not correlate with the actual resistance status of tested populations, indicating that tested polymorphisms are not tightly correlated with the resistance in T. urticae. Authors confirm the utility of TaqMan to detect mutations in T. urticae and further demonstrate that it can be used for field-collected populations.

The information presented is useful and is adding to several attempts to develop diagnostic tools to predict pesticide resistance in mite populations based on genotype information. The main challenges there is not necessarily detection of the allelic frequency, but their correlation with the resistance phenotype. Next, what are cut offs for defining a population as resistant vs susceptible based on allele frequency, even if it is 100% predictive of the resistance. Manuscript would benefit from additional data, explanations and some corrections.

We agree with the reviewer that cutoff currently is not well defined and would be hard for a grower to determine when to consider a population as resistant. This is one of the fundamental reasons why we composed this manuscript to demonstrate this problem. “However, how a grower would interpret the results in the context of pest management decisions needs to be further addressed.”

a) Line 221: Why did authors choose bifenazate (G126S), see Xue W, Wybouw N, Van Leeuwen T. The G126S substitution in mitochondrially encoded cytochrome b does not confer bifenazate resistance in the spider mite Tetranychus urticae. Experimental and Applied Acarology. 2021 Dec;85(2):161-72. At least they should detect S141F SNP as well, as per DOI:10.1038/s41598-017-09054-y.

We agree with the reviewer that G126S is not a good indicator of resistance. We have addressed this in-depth throughout the manuscript. We feel that findings of this manuscript support the work that was done by Xue et al. 2021. All of our work was conducted before Xue et al. manuscript was published and feel how it is written is appropriate and supports their findings.

Likewise, what was the rationale to detect VGSC (F1538I) when it was reported to be a poor predictor of mite resistance to bifenthrin?

We used the F1538I gene due to the extensive research that revolve around this target candite. The targets that we choose were limited. In order for TaqMan qPCR to be a practical tool for growers, all known targets will have to have primer and probes designed for them.

chs (I1017F) should be tightly correlated to etoxazole resistance.

b) Line 250: “The TaqMan assay predicted population 2 (collected from hops in Washington) as a completely resistant population when, in fact, there was a high proportion of wildtype alleles (17.45%). As such, the population should be considered heterozygous.” Authors refer to Etoxazole data? They should clearly state it. In addition, they should define criteria for calling populations heterozygous/homozygous, and in particular resistant vs sensitive. Population 2 should give rise to 68% of mites homozygous to I1017F SNP and thus predicted to be resistant. Is this population resistant or susceptible according to authors? What should be cut offs and how are they defined?

The reviewer brings up an important point of cutoffs for wildtype and resistance phenotypes. The authors used Bio-Rad CFX Maestro software to classify TaqMan based results into homozygous wildtype, heterozygous, and homozygous resistance. We have clarified this in the methods. We confirmed the results of the TaqMan assay with a Multiplex PCR. The cutoff that we used to determine resistance using the Multiplex reaction was that wildtype or resistant population has to have at least 90% of the reads to be confirmed. We noted that this information was not included in the manuscript and have now included it.

c) Line 259:

Median lethal dose assay was performed only for bifenazate resistance. As per Xue W, Wybouw N, Van Leeuwen T. The G126S substitution in mitochondrially encoded cytochrome b does not confer bifenazate resistance in the spider mite Tetranychus urticae. Experimental and Applied Acarology. 2021 Dec;85(2):161-72., this is the least predictable association. Authors concur in line 269, but they should have known it ahead of the time.

Xue et al. Manuscript was published in December of 2021. The data for this manuscript was collected and processed before that manuscript was published. The findings of this manuscript further support their results.

Authors should add etoxazole bioassays.

We agree that additional bioassays would be beneficial to this study. However, at this time we are unable to perform additional assays (please refer to our cover letter). We believe the findings of the investigation are still relevant.

d) Line 284-297: “Our findings confirm the utility of TaqMan to detect mutations in T. urticae and further demonstrates that it can be used for field-collected populations.” “However,

how a grower would interpret the results in the context of pest management decisions needs to be further addressed.” Authors have to define or at least propose how TaqMan data can be used for diagnostic decision.

To discuss diagnostic decisions we have added, “However, how a grower would interpret the results in the context of pest management decisions needs to be further addressed. However, how a grower would interpret the results in the context of pest management decisions needs to be further addressed. One suggestion is that the grower could make a decision regarding chemical application based on how fixed the genotype is in the field. Fields that are homozygous susceptible or homozygous resistant pose little concern for determining if a pesticide application should be made, as susceptible fields could be treated while the resistant fields should not be. The harder decisions come when fields are heterozygous. Future investigations are required in order to make this technique useful in those management decisions.”

e) Line 321: “A pyrethroid (bifenthrin) has never been registered for use on mint in the Pacific Northwest. The TaqMan qPCR and targeted-sequencing data for F1538I (Bifenthrin) suggested that most mint field populations of mites have some proportion of a resistance phenotype for Bifenthrin.”

Careful!!! There was no resistance bioassay performed. According to DOI:10.1038/s41598-017-09054-y, this SNP is not very predictive of the resistance state.

Thank you for the correction, we changed phenotype to genotype.

f) Line 339: “To confirm the results of the genotypic assays, we performed an LD50 assessment on bifenazate resistance. We decided to confirm resistance to one of the chemicals we explored, bifenazate, which can be predicted with the mutation G126S in the cytochrome b gene.” This contradicts data presented and data in DOI:10.1038/s41598-017-09054-y and Xue et al., 2021.

We go into significant detail regarding that G126S is not a good marker for resistance. This is mentioned throughout the manuscript including, “To confirm the results of the genotypic assays, we performed an LD^50^ assessment on bifenazate resistance. We decided to confirm resistance to one of the chemicals we explored, bifenazate, which can be predicted with the mutation G126S in the cytochrome b gene. The genotypic data suggested that *T. urticae* collected from hop yards and mint fields from Washington would demonstrate different resistant phenotypes. The LD50 assessment for *T. urticae* collected from mint fields demonstrated a higher LD50 value than *T. urticae* collected from hop yards. The results of the LD50 study suggested the exact opposite of the genetic data, with populations collected from mint fields demonstrating slightly higher bifenazate resistance than hop yards. Recent studies agree with our findings and suggest that the G126S mutation may not be a good indicator that a population is resistant to bifenazate (24). It is likely that multiple markers will be required to accurately detect resistance in field populations and further that the G126S mutation is not an optimal indicator of resistance status.”

Corrections:

Grammar in sentences in lines: 86-88, 99-100

We have reworded these lines.
