# Supplementary material for: Using targeted sequencing and TaqMan approaches to detect acaricide (bifenthrin, bifenazate, and etoxazole) resistance associated SNPs in Tetranychus urticae collected from peppermint fields and hop yards
Source: PLoS One. 2023 Mar 23;18(3):e0283211. doi: 10.1371/journal.pone.0283211 (PMC10035822; doi:10.1371/journal.pone.0283211)
Supplement: Attachment — Submitted filename: Review report PONE-D-22-09155_R1.docx [file pone.0283211.s004.docx]

Review report PONE-D-22-09155R1: Detection of bifenthrin, bifenazate, etoxazole resistance in *Tetranychus urticae* collected from peppermint fields and hop yards using targeted sequencing and TaqMan approaches in pooled DNA samples

The authors examined the use of a TaqMan qPCR-based approach to determine acaricide resistance genotypes in field-collected populations of *T. urticae* from peppermint fields and hop yards in the Pacific Northwest of the United States and confirmed the results with a multiplex targeted sequencing. Although the MS is improved much, the following point need to be addressed.

The authors changed the title as ‘Using targeted sequencing and TaqMan approaches to detect acaricide resistance associated SNPs in *Tetranychus urticae* collected from peppermint fields and hop yards’

I appreciate their decision and would suggest specifying the acaricide name(s) in the title rather acaricide. Still, I am not convinced that without examining the median lethal dose assay of bifenthrin and etoxazole to detect the acaricide resistance associated SNPs. If this is not possible to perform these studies in this stage, that should be addressed in the discussion.
