# Supplementary material for: Using targeted sequencing and TaqMan approaches to detect acaricide (bifenthrin, bifenazate, and etoxazole) resistance associated SNPs in Tetranychus urticae collected from peppermint fields and hop yards
Source: PLoS One. 2023 Mar 23;18(3):e0283211. doi: 10.1371/journal.pone.0283211 (PMC10035822; doi:10.1371/journal.pone.0283211)
Supplement: Attachment — Submitted filename: Response to Reviewers 11_16_22.docx [file pone.0283211.s005.docx]

Cover Letter

Dear Dr. Hamim and Reviewers

Thank you very much for your review and consideration of our manuscript “PONE-D-22-09155, Title: Using targeted sequencing and TaqMan approaches to detect acaricide resistance associated SNPs in Tetranychus urticae collected from peppermint fields and hop yards”, submitted for review in PLOS ONE. We understand the amount of work and effort that the editor and reviewers have taken to improve this manuscript and appreciate your feedback and comments.

We took the comments from the reviewers very seriously. The comments from the reviewers and the editor were helpful in improving our manuscript. Please find enclosed our corrections and comments to reviewers 4 and 6, on a point-by-point basis. Reviewer comments are in font color black and response to reviewers’ comments are in blue. We feel this manuscript has benefited from the peer-review process and look forward to any further suggestions for improvement if they arise.

Sincerely,

The Authors

Justin Clements (corresponding author), Ph.D.

[Jclements3323@gmail.com](mailto:Jclements3323@gmail.com)

Reviewer #4:

The authors examined the use of a TaqMan qPCR-based approach to determine acaricide resistance genotypes in field-collected populations of *T. urticae* from peppermint fields and hop yards in the Pacific Northwest of the United States and confirmed the results with a multiplex targeted sequencing. Although the MS is improved much, the following point need to be addressed.

The authors changed the title as ‘Using targeted sequencing and TaqMan approaches to detect acaricide resistance associated SNPs in Tetranychus urticae collected from peppermint fields and hop yards’

I appreciate their decision and would suggest specifying the acaricide name(s) in the title rather acaricide.

We have changed the name of the manuscript to include the acaricides that we investigated.

Still, I am not convinced that without examining the median lethal dose assay of bifenthrin and etoxazole to detect the acaricide resistance associated SNPs. If this is not possible to perform these studies in this stage, that should be addressed in the discussion.

We thank the reviewer for their comment. We have further addressed this in the discussion of the manuscript, “In the future, all SNPs being considered for the detection of acaricide resistance using a TaqMan approach should be validated through LD_50_ assessments for each specific acaricides. In this investigation we were only able to conduct an LD_50_ assessment with bifenazate and, as such, further investigations should be conducted to confirm that the genotyping data matches resistance phenotypes for bifenthrin and etoxazole.”

Reviewer #6:

I find that revisions did not address the main problem of this manuscript. Yes, authors demonstrated that TaqMan protocol can identify SNPs in mite populations, BUT, authors do not state clearly that we do not have genetic markers for mite pesticide resistance (except etoxazole based on the prior research). Instead, the revised manuscript states in the abstract, line 33: “Within this investigation we examined the use of a TaqMan qPCR-based approach to determine acaricide resistance genotypes in field-collected populations of T. urticae from peppermint fields and hop yards in the Pacific Northwest of the United States and confirmed the results with a multiplex targeted sequencing.” This is MISLEADING! Authors detected SNPs (genotypes), but not acaricide resistance.

It is not our intention to be misleading in any form. In the manuscript we address that SNPs have been classified through peer reviewed literature which have implicated them with resistance phenotypes. We have changed this section to read as, “Within this investigation we examined the use of a TaqMan qPCR-based approach to determine genotypes which have been **previously associated** with acaricide resistance in field-collected populations of *T. urticae* from peppermint fields and hop yards in the Pacific Northwest of the United States and confirmed the results with a multiplex targeted sequencing.”

Furthermore, in Introduction authors state,

Line 81: SNPs in multiple, different genes have become associated with the development of resistance to different acaricide chemistries including bifenthrin, bifenazate, and etoxazole (17-20). Historically these mutations have been detected through DNA sequencing that can be analyzed for the presence of the polymorphism. However, multiple investigations have recently suggested using these polymorphisms as a diagnostic tool to inform agricultural growers of the acaricide resistance status of individual field populations of T. urticae. This would allow the grower to make an informed decision into which acaricide would adequately control their local T. urticae populations and may prevent further resistance development (21,22). Three of the most common polymorphisms studied are a mutation that results in the amino acid change. The first results in a change of a glycine to a serine in the cytochrome b gene (G126S) in the mitochondrial respiratory chain. This mutation has been shown to confer resistance to bifenazate, a carboxylic ester, in T. urticae (23). The effects of the G126S mutation have recently been called into question and new research suggests that this mutation cannot be used to predict acaricide resistance (24). Another mutation at amino acid 1538 in the voltage gate channel gene results in a phenylalanine changing to an isoleucine. This mutation confers resistance to bifenthrin, a pyrethroid (16) and has also been shown to confer resistance to the growth inhibitors, hexythiazox and clofentezine (25). Finally, a mutation in the chitin synthase 1 gene that changes an isoleucine to a phenylamine has been shown to result in resistance to etoxazole, a narrow spectrum systemic acaricide (20). These mutations have been characterized by sequencing both susceptible and resistant populations of T. urticae and have provided in-depth information on the development of resistance to acaricides in T. urticae (16-20, 23). A restriction digest has also been used to screen mite populations for the I1017F mutation(26).”

In line 93, authors provide information that G126S substitution is not predictive of mite resistance to Bifenazate. But they do not provide such disclaimer for F1538I (Bifenthrin)!!

We thank the reviewer for the comment and we want to make sure that the manuscript is accurately written and appreciate the feedback.

We have included the citation, “Riga M, Bajda S, Themistokleous C, Papadaki S, Palzewicz M, Dermauw W, Vontas J, Leeuwen TV. The relative contribution of target-site mutations in complex acaricide resistant phenotypes as assessed by marker assisted backcrossing in *Tetranychus urticae*. Scientific reports. 2017 Aug 23;7(1):1-2.” within the introduction and discussion. The discussion now reads: “The mutation F1538I, which has been previously associated with bifenthrin resistance, has also been suggested to be a poor predictor of resistance phenotypes (26). In the future, all SNPs being considered for the detection of acaricide resistance using a TaqMan approach should be validated through LD_50_ assessments for each specific acaricides. In this investigation we were only able to conduct an LD_50_ assessment with bifenazate and, as such, further investigations should be conducted to confirm that the genotyping data matches resistance phenotypes for bifenthrin and etoxazole.”

The reason why we choose to examine this mutation (F1538I) was the depth of information that implicate this mutation in resistance phenotypes in the Pacific Northwest. Including recent investigation:

Piraneo TG, Bull J, Morales MA, Lavine LC, Walsh DB, Zhu F. Molecular mechanisms of Tetranychus urticae chemical adaptation in hop fields. Scientific reports. 2015 Dec 1;5(1):1-2.

Wu M, Adesanya AW, Morales MA, Walsh DB, Lavine LC, Lavine MD, Zhu F. Multiple acaricide resistance and underlying mechanisms in Tetranychus urticae on hops. Journal of Pest Science. 2019 Mar;92(2):543-55.

Adesanya AW, Lavine MD, Moural TW, Lavine LC, Zhu F, Walsh DB. Mechanisms and management of acaricide resistance for Tetranychus urticae in agroecosystems. Journal of Pest Science. 2021 Jun;94(3):639-63.

I understand that this research has been done while some of the relationships between SNAs and resistance status were not available. However, the paper is written NOW and has to position facts correctly.

We appreciate the reviewers comment as we want to make sure that the manuscript is appropriate and accurately written, throughout the manuscript (abstract, introduction and discussion) we address new findings regarding G126S, including the findings of this research which further confirms Xue et al. 2021 research. We further have added a section regarding the ability to use F1538I as a predictor of resistance within the manuscript.

Line 41: While we were able to detect the SNPs associated with the examined acaricides, the mutation G126S was not an appropriate or accurate indicator for bifenazate resistance.

Line 93: The effects of the G126S mutation have recently been called into question and new research suggests that this mutation cannot be used to predict acaricide resistance (24). Another mutation at amino acid 1538 in the voltage gate channel gene results in a phenylalanine changing to an isoleucine. This mutation has been suggested to confer resistance to bifenthrin, a pyrethroid (16) and has also been shown to confer resistance to the growth inhibitors, hexythiazox and clofentezine (25). However, some studies bring into question the accuracy of F1538I to predict bifenthrin resistance (26).

Line 373: Recent studies agree with our findings and suggest that the G126S mutation may not be a good indicator that a population is resistant to bifenazate (24). It is likely that multiple markers will be required to accurately detect resistance in field populations and further that the G126S mutation is not an optimal indicator of resistance status. The mutation F1538I, which has been previously associated with bifenthrin resistance, has also been suggested to be a poor predictor of resistance phenotypes (26). In the future, all SNPs being considered for the detection of acaricide resistance using a TaqMan approach should be validated through LD_50_ assessments for each specific acaricides. In this investigation we were only able to conduct an LD_50_ assessment with bifenazate and, as such, further investigations should be conducted to confirm that the genotyping data matches resistance phenotypes for bifenthrin and etoxazole.
