# Supplementary material for: Using targeted sequencing and TaqMan approaches to detect acaricide (bifenthrin, bifenazate, and etoxazole) resistance associated SNPs in Tetranychus urticae collected from peppermint fields and hop yards
Source: PLoS One. 2023 Mar 23;18(3):e0283211. doi: 10.1371/journal.pone.0283211 (PMC10035822; doi:10.1371/journal.pone.0283211)
Supplement: Attachment — Submitted filename: Response to Reviewers 2_7_23.docx [file pone.0283211.s006.docx]

Cover Letter

Dear Dr. Hamim and Reviewers

Thank you very much for your review and consideration of our manuscript “PONE-D-22-09155, Title: Using targeted sequencing and TaqMan approaches to detect acaricide (bifenthrin, bifenazate, and etoxazole) resistance associated SNPs in Tetranychus urticae collected from peppermint fields and hop yards”, submitted for review in PLOS ONE. We understand the amount of work and effort that the editor and reviewers have taken to improve this manuscript and appreciate your feedback and comments.

We took the comments from the reviewers very seriously. The comments from the reviewers and the editor were helpful in improving our manuscript. Please find enclosed our corrections and comments to reviewer 6, on a point-by-point basis. Reviewer comments are in font color black and response to reviewers’ comments are in blue. We feel this manuscript has benefited from the peer-review process and look forward to any further suggestions for improvement if they arise.

Sincerely,

The Authors

Justin Clements (corresponding author), Ph.D.

[Jclements3323@gmail.com](mailto:Jclements3323@gmail.com)

Reviewer 6:

1) The effort to modify the manuscript is appreciated. However, authors have to go over manuscript and systematically change parts that refer to analysed SNPs as a tool for detection of pesticide resistance in mite populations. Authors state:” In the present study, we set out to investigate whether we could use a pooled TaqMan qPCR approach to monitor resistant populations of T. urticae. We chose to examine a limited set of three well defined markers G126S (Cytochrome b), F1538I (Voltage gated sodium channel), and I1017F (Chitin synthase 1) using a pooled TaqMan approach.” This is a good rationale for the study and is independent of utility of these SNPs for detection of mite pesticide resistance. Reading the manuscript, authors are continuously trying to justify the choice of SNPs and to link it to the resistance.

Sorry for the insistence, but one should clearly state the purpose of the work and should critically state the utility of analysed SNPs as being diagnostic for mite pesticide resistance. Already in the field, there is confusion of whether they are usable or not. Authors themselves have chosen these SNPs based on initial publications that suggested that they can be diagnostic.

We agree with the reviewer that the focus of the manuscript should be clearly stated through out the manuscript. We have clarified throughout the manuscript that we are specifically detecting resistance associated SNPs and not detecting resistance.

2) This manuscript should not continue to propagate this misconception. For example running title: Detection of acaricide resistance in Tetranychus urticae using molecular based approaches” is not appropriate and should be changed.

We have changed the running title from, “Detection of acaricide resistance in *Tetranychus urticae* using molecular based approaches” to, “Detection of acaricide resistance associated SNPs in *Tetranychus urticae*”

3) Also, authors introduced modifications as:” This mutation has been suggested to confer resistance to bifenthrin (please add REF), a pyrethroid (16) and has also been shown to confer resistance to the growth inhibitors, hexythiazox and clofentezine (25). However, some studies bring into question the accuracy of F1538I to predict bifenthrin resistance (26).” Can authors make a single statement in the introduction that will critically establish the utility of reported SNPs as diagnostic? The fact that most of them are not, is not diminishing their work.

We have added the following section to the introduction, “Additionally, recent findings indicate that some of the polymorphisms associated with resistance may not be useful as a diagnostic tool, including G126S in the cytochrome b gene and F1538I in the voltage gate channel (24,26).” We also hope to convey this point with the following statements in the discussion “In the future, all SNPs being considered for the detection of acaricide resistance using a TaqMan approach should be validated through LD50 assessments for each specific acaricides.” and “While these genetic approaches are constantly evolving based on our knowledge of putative and nearby polymorphisms (crucial for a stable assay), further work is needed to validate the importance of specific mutations and the metrics used for determining overall resistance profiles within a population before they can be used to inform pest management decisions.”

4) Please have a careful reading of the manuscript and eliminate some grammar mistakes.

The authors have gone through the manuscript for grammar and edited manuscript to the best of our ability.
